# Brain MRI CO_2_ Stress Testing: A Pilot Study in Patients with Concussion

**DOI:** 10.1371/journal.pone.0102181

**Published:** 2014-07-17

**Authors:** W. Alan C. Mutch, Michael J. Ellis, M. Ruth Graham, Vincent Wourms, Roshan Raban, Joseph A. Fisher, David Mikulis, Jeffrey Leiter, Lawrence Ryner

**Affiliations:** 1 Department of Anesthesia and Perioperative Medicine, Health Sciences Centre, University of Manitoba, Winnipeg, Manitoba, Canada; 2 Department of Surgery, Section of Neurosurgery, Health Sciences Centre, University of Manitoba, Winnipeg, Manitoba, Canada; 3 Department of Anesthesia and Pain Management, Toronto General Hospital, University of Toronto, Toronto, Ontario, Canada; 4 Department of Radiology, Section of Neuroimaging, Toronto Western Hospital, University of Toronto, Toronto, Ontario, Canada; 5 Department of Surgery, Pan Am Clinic, University of Manitoba, Winnipeg, Manitoba, Canada; 6 Department of Physics and Astronomy, University of Manitoba, Winnipeg, Manitoba, Canada; Hangzhou Normal University, China

## Abstract

**Background:**

There is a real need for quantifiable neuro-imaging biomarkers in concussion. Here we outline a brain BOLD-MRI CO_2_ stress test to assess the condition.

**Methods:**

This study was approved by the REB at the University of Manitoba. A group of volunteers without prior concussion were compared to post-concussion syndrome (PCS) patients – both symptomatic and recovered asymptomatic. Five 3-minute periods of BOLD imaging at 3.0 T were studied – baseline 1 (BL1– at basal CO_2_ tension), hypocapnia (CO_2_ decreased ∼5 mmHg), BL2, hypercapnia (CO_2_ increased ∼10 mmHg) and BL3. Data were processed using statistical parametric mapping (SPM) for 1^st^ level analysis to compare each subject’s response to the CO_2_ stress at the p = 0.001 level. A 2^nd^ level analysis compared each PCS patient’s response to the mean response of the control subjects at the p = 0.05 level.

**Results:**

We report on 5 control subjects, 8 symptomatic and 4 asymptomatic PCS patients. Both increased and decreased response to CO_2_ was seen in all PCS patients in the 2^nd^ level analysis. The responses were quantified as reactive voxel counts: whole brain voxel counts (2.0±1.6%, p = 0.012 for symptomatic patients for CO_2_ response < controls and 3.0±5.1%, p = 0.139 for CO_2_ response > controls: 0.49±0.31%, p = 0.053 for asymptomatic patients for CO_2_ response < controls and 4.4±6.8%, p = 0.281 for CO_2_ response > controls).

**Conclusions:**

Quantifiable alterations in regional cerebrovascular responsiveness are present in concussion patients during provocative CO_2_ challenge and BOLD MRI and not in healthy controls. Future longitudinal studies must aim to clarify the relationship between CO_2_ responsiveness and individual patient symptoms and outcomes.

## Introduction

Concussion is a form of traumatic brain injury (TBI) caused by biomechanical forces imparted to the head resulting in alterations in neurological functioning with or without a loss of consciousness. It is estimated that 1.6–3.8 million sports-related concussions occur in the United States per year. [1] Recognition and diagnosis of concussion remains challenging due to the variability in clinical manifestations and symptoms, patient under-reporting, and a persistent lack of knowledge about this condition amongst participating athletes, parents, and even treating physicians. [2] [3] [4] In recent years, physician management of concussion has benefitted from the development of standardized concussion symptom inventories and computerized neuro-cognitive testing tools. [5] [6] [7] [8] None of these tools provide a window into the pathophysiology of concussion. In contrast, functional magnetic resonance imaging (fMRI) with blood oxygen level-dependent (BOLD) echo-planar imaging (EPI) has revolutionized our understanding of the brain at ‘work’ and at ‘rest’. [9] Deep insights into cognition, consciousness and disease states have resulted from increasingly sophisticated post-hoc analysis of BOLD EPI signals under ‘task’ and ‘no task’ conditions. [10] [11] [12] Numerous studies have examined fMRI activation patterns in patients with concussion during cognitive tasks that activate specific neural pathways and brain regions. However, none of these studies have applied a global physiological stimulus as a ‘task’ in an effort to assess the cerebrovascular integrity of the entire brain. Although alterations in the cerebrovascular response to CO_2_ challenge have been demonstrated in TBI and stroke, these parameters have not been previously studied in patients with concussion.

Here we describe a novel neuro-imaging approach that allows global assessment of whole-brain cerebrovascular responsiveness to CO_2_ challenge – an MRI CO_2_ stress test of the brain. This stress test uses model-based prospective end-tidal targeting (MPET) of CO_2_ while measuring the regional BOLD response on cerebral oxygenation resulting from the CO_2_ stimulus during MRI in healthy volunteers and patients with concussion. Individual and group differences in regional responsiveness to CO_2_ stress testing were evaluated using post-hoc statistical parametric mapping (SPM) and analysis, a well established method of analyzing fMRI data [13] [14].

## Methods

The study was approved by the Biomedical Research Ethics Board (BREB) of the University of Manitoba. Concussion patients were recruited from the Pan Am Concussion Program at the Pan Am Clinic in Winnipeg MB. All subjects gave written, witnessed, informed consent on the BREB approved consent form on the day of their study. All subjects were also independently screened by the MR technologist as to compatibility for MRI and signed this consent form as well. Imaging was undertaken at the Kleysen Institute for Advanced Medicine (KIAM) at the Health Sciences Centre in Winnipeg. All concussion patients were evaluated by a single neurosurgeon (M.E.) who confirmed the diagnosis of concussion according to established criteria. [8] All study participants completed the post concussion symptom scale (PCSS) – a 22-item symptom inventory that generates a severity score out of a total of 132 points. Concussion patients were stratified into two groups – a “symptomatic group” who suffered a concussion in the past 12 months, remained symptomatic since the time of injury and who displayed a PCSS>5 points and an “asymptomatic group” who had a history of previous concussions but were now recovered, and who displayed a PCSS<5 points. Clinical history was also obtained for all control subjects. None of the control subjects had a past medical history significant for TBI. One subject had experienced a single concussion over a decade earlier with no residua. Demographic, past medical and concussion histories for the control subjects and concussion patients were compared. ANOVA tables were generated to compare hemodynamic and CO_2_ response data between groups; p<0.05 considered statistically significant.

### Neuro-imaging assessment

The neuro-imaging assessment was divided into two testing stages: a pre-imaging testing stage and an MRI testing stage.

#### i) Pre-imaging Study

During the pre-imaging testing stage the control subjects and patients underwent non-invasive model-based prospective end-tidal (MPET) CO_2_ targeting during infrared cerebral oximetry and hemodynamic monitoring. The individual’s age, height and weight and gender were collected and entered into a computer that interfaced with the MPET device (RespirAct, Thornhill Research Inc.) to determine baseline O_2_ consumption and CO_2_ production formulated on established algorithms. [15] The subject donned a tight-fitting facemask attached to a custom-designed breathing circuit developed for the MPET device. In this way controlled end-tidal gas mixtures could be reproducibly administered allowing precisely controlled step-changes in end-tidal CO_2_ under isoxic conditions. Using the patient’s ETCO_2_ as baseline, a patient-specific, provocative CO_2_ breathing sequence was designed. End-tidal O_2_ was targeted at 115 mmHg throughout while end-tidal CO_2_ was targeted in five successive 3-minute intervals – baseline values (BL1), hypocapnia (∼5 mmHg below BL1), BL2, hypercapnia (∼10 mmHg above BL1) and BL3. During the provocative breathing sequence, continuous bifrontal cerebral oximetry was measured using two near infrared sensors (Fore-Sight, CasMed Inc., Hartford, CT). During the same period, non-invasive hemodynamic monitoring of heart rate (HR) and blood pressure (BP) was conducted at 3-minute intervals. To ensure tolerability and assess concussion symptoms throughout the testing stage, the patient was instructed to rate their concussion symptoms on a four-point scale every minute on the following scale: 0 = no symptoms, 1 = mild, 2 = moderate, 3 = severe. If the patient tolerated the pre-imaging testing stage, they were given the opportunity to participate in the MRI testing stage of the study.

#### ii) MRI Study

During the MRI testing stage the control subjects and patients underwent the same MPET CO_2_ targeting sequence with hemodynamic monitoring and MRI using a Siemens Verio 3 tesla (3.0 T) MRI scanner with a 12-channel head coil. The imaging sequences comprised a localizer, a B0-field map, a T1-weighted MPRAGE, and BOLD EPI. The BOLD sequence parameters were: FOV 240×240 mm, 20 slices, slice thickness 5 mm, interslice gap 2 mm, TR 2000 ms, TE 30 ms, flip angle 85°, voxel size 3.8×3.8×5.0 mm. During the BOLD sequence the above described CO_2_ challenge test was utilized.

### Preprocessing of MRI sequences

Standard preprocessing of the MRI EPI output was accomplished for the BOLD sequence with SPM8 software, including batch processing by an SPM toolbox. The preprocessing included re-alignment of images, slice time correction, co-registration with the MPRAGE images, smoothing and normalization into MNI space. Studies were rejected if motion was greater than 3 mm in any plane.

### 1^st^ Level Analysis

First level analyses comparing the hypercapnic to hypocapnic sequences were undertaken for each study participant. The CO_2_ response of the control subject group was compared to concussion patient values. Images were assessed with a grey and white matter inclusive mask applied with significance set at the p<0.001 uncorrected level.

### 2^nd^ Level Analysis

A second level analysis for the BOLD studies was undertaken with data from the control subjects combined into an atlas allowing data from each concussion patient to be compared to control mean values. In this comparison, regions where BOLD signal was less than and greater than the anticipated cerebrovascular response (CVR) established by the control subject group means were examined. The voxel counts:whole brain voxel count ratios for each symptomatic and asymptomatic concussion patients for the response less than and greater than the atlas of control subjects were calculated. ANOVA tables were generated to compare the voxel count ratios between groups; p<0.05 considered statistically significant. If significant, comparison between individual groups was undertaken. These count ratios were compared to the voxel count ratios from the pooled control atlas values by unpaired t-test for unequal variances with p<0.05 in the two-tailed test considered statistically significant. The 2^nd^ level results for voxel counts for each symptomatic concussion patient at the p<0.05 level (both less than and greater than when compared to the control subjects group mean) were also compared to the PCSS values for each patient by linear regression.

## Results

A total of 30 control subjects/patients were studied. Individuals were excluded from analysis for the following reasons – protocol violations with incomplete BOLD sequences – 2 individuals; falling asleep while in the magnet resulting in poor end-tidal gas targeting – 3 individuals; excessive movement – 3 individuals; claustrophobia – 2 individuals; poor mask fit resulting in poor end-tidal gas targeting – 1 individual; custom mixed gas delivery failure resulting in non-targeting of end-tidal gases – 1 individual; patient identified post- hoc with serious psychiatric history on medication – 1 individual. We report on 5 control subjects and 12 concussion patients, 8 symptomatic patients (mean PCSS = 28±16; range 5–51) and 4 asymptomatic patients (mean PCSS = 1; range 0–2).

Study participant demographic, past medical and concussion histories, presenting symptoms, and PCSS scores are summarized in Table 1.

**Table 1:** Demographics.

	Subject	Study#	Age/Gender	#PreviousConcussions/PMHx	Mechanism	Duration ofSymptoms	Symptoms	PCSS attime ofstudy
**Controls**	1	C004	27M	0	N/A	N/A	N/A	0
	2	C008	39M	0	N/A	N/A	N/A	0
	4	C012	41M	0	N/A	N/A	N/A	0
	5	C013	33M	remote>10 years prior	N/A	N/A	N/A	0
	6	C014	29M	0	N/A	N/A	N/A	0
**Symptomatic** **PCS**	1	mTBI004	39M	none,migraine	sport-cycling	6 months	migraine headache, photo,gaze instability	39
	2	mTBI006	41M	4	MVA	2 months	cognitive, sleep disturbance,affective symptoms,visual disturbance	51
	3	mTBI008	38F	7	swimming accident	2 months	exertional headache, posturalinstability, affective	12
	4	mTBI009	21M	3	sport-football	2 months	exertional headache, photo,phono, cognitive	5
	5	mTBI010	46M	none, remoteviral meningitis	sport-road skiing	2 months	headache, gaze instability,postural instability	34
	6	mTBI012	26M	4	fall on ice	3 weeks	headache, cognitive, fatigue,sleep disturbance	19
	7	mTBI013	35M	none,Addison'sdisease	sport-hockey	2 weeks	headache, postural instability,dizziness	22
	8	mTBI014	34M	1	school accident	1 month	headache, photo,cognitive, sleep	42
**Asymptomatic** **PCS**	1	mTBI002	23M	4	sport-football	asymptomatic	asymptomatic	2
	2	mTBI003	19M	3	sport-hockey	9 months	blurred vision	0
	3	mTBI011	37M	4	sport-cycling	12 months	headache, photo	2
	4	mTBI016	21M	4	sport-hockey	asymptomatic	asymptomatic	0

**Figure 1:**
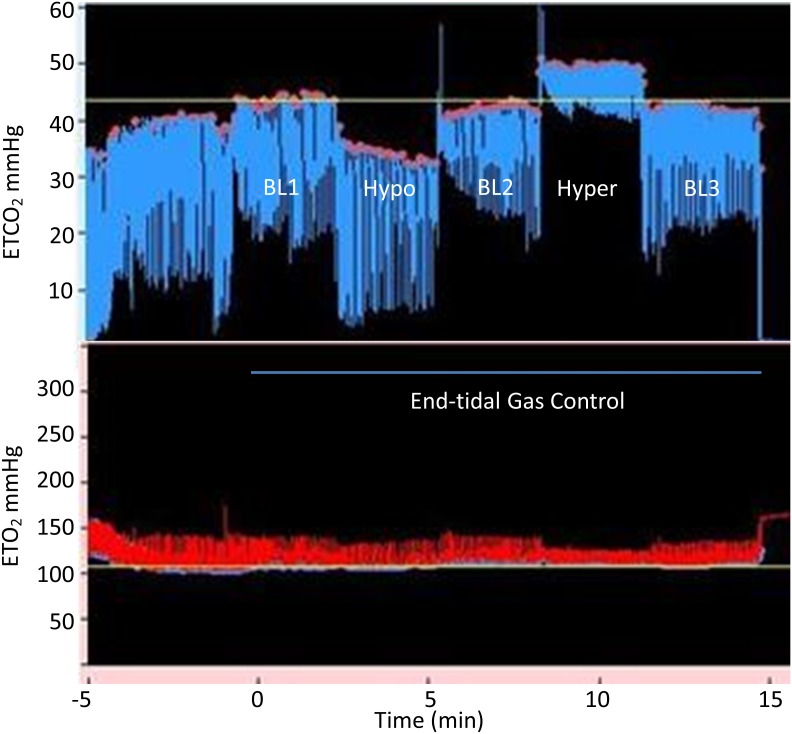
An example of the end-tidal gas control with the MPET device. The end-tidal CO_2_ controlled response is shown in the upper panel. The period of time when the MPET device was active is shown by the horizontal line below. This occurred while the patient was undergoing BOLD EPI. The breathing sequence at baseline 1 (BL1), hypocapnia (Hypo), BL2, hypercapnia (Hyper) and BL3 is seen. In the lower panel the end-tidal O_2_ trace is seen. The stable end-tidal values during the period of MPET control can be seen.

**Figure 2:**
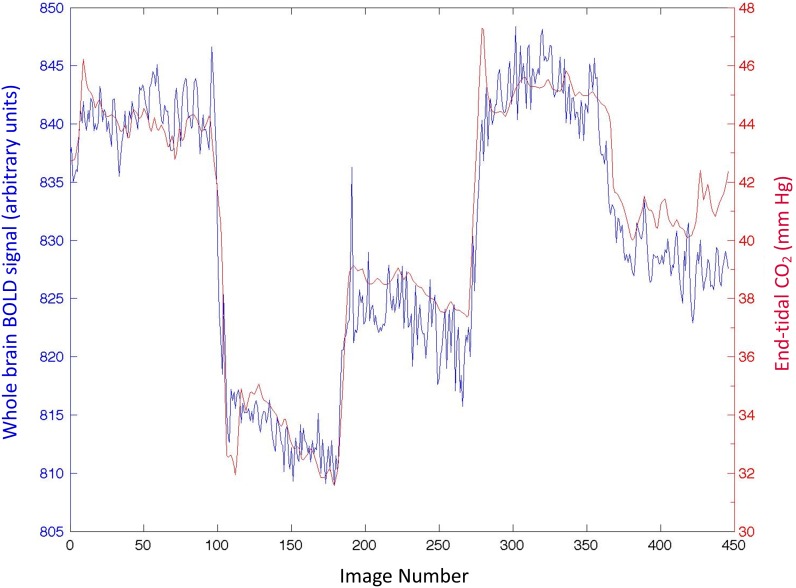
The relationship between whole brain BOLD EPI signal and end-tidal CO_2_ as controlled by the MPET device. The R^2^-value for the regression between the two signals was 0.93 in this instance.

Hemodynamic responses during the preliminary cerebral saturation study and during BOLD imaging are shown in Table 2. The CO_2_ delta (mean breath-by-breath difference for the hypercapnic period – the hypocapnia period) was 9.2±1.4 mmHg in the control group and 11.5±1.9 mmHg in the symptomatic PCS group and 11.6±2.4 mmHg for the asymptomatic concussion group (p = 0.114 ANOVA between groups). The normalized CVR to CO_2_ for each subject is also shown here as are the change in mean blood pressure from the hypocapnic to the hypercapnic period. An example of the end-tidal targeting with the MPET device is shown in Figure 1. Comprehensive results relating to end-tidal gas control and cerebral oximetry will be published separately. An example of the tight relationship between whole brain BOLD signal with the MPET-induced changes in end-tidal CO_2_ is shown in Figure 2.

**Table 2:** Hemodynamics and End-tidal Gases.

	Controls	Sym PCS	Asym PCS	p-values
Blood Pressure Change	7.2±6.1	7.1±6.7	12.3±10.5	0.504
Baseline end-tidal O_2_	108.3±2.4	111.0±5.0	108.5±3.5	0.443
Baseline end-tidal CO_2_	38.7±3.3	40.1±3.5	40.1±1.7	0.71
CO_2_ Change	9.2±1.6	11.5±1.9	11.6±2.4	0.114

PCS-post-concussion syndrome.

Sym-symptomatic.

Asym-asymptomatic.

End-tidal tensions in mmHg.

Breath-by-breath change in CO_2_ between hypercapnia and hypocapnia in mmHg.

**Figure 3:**
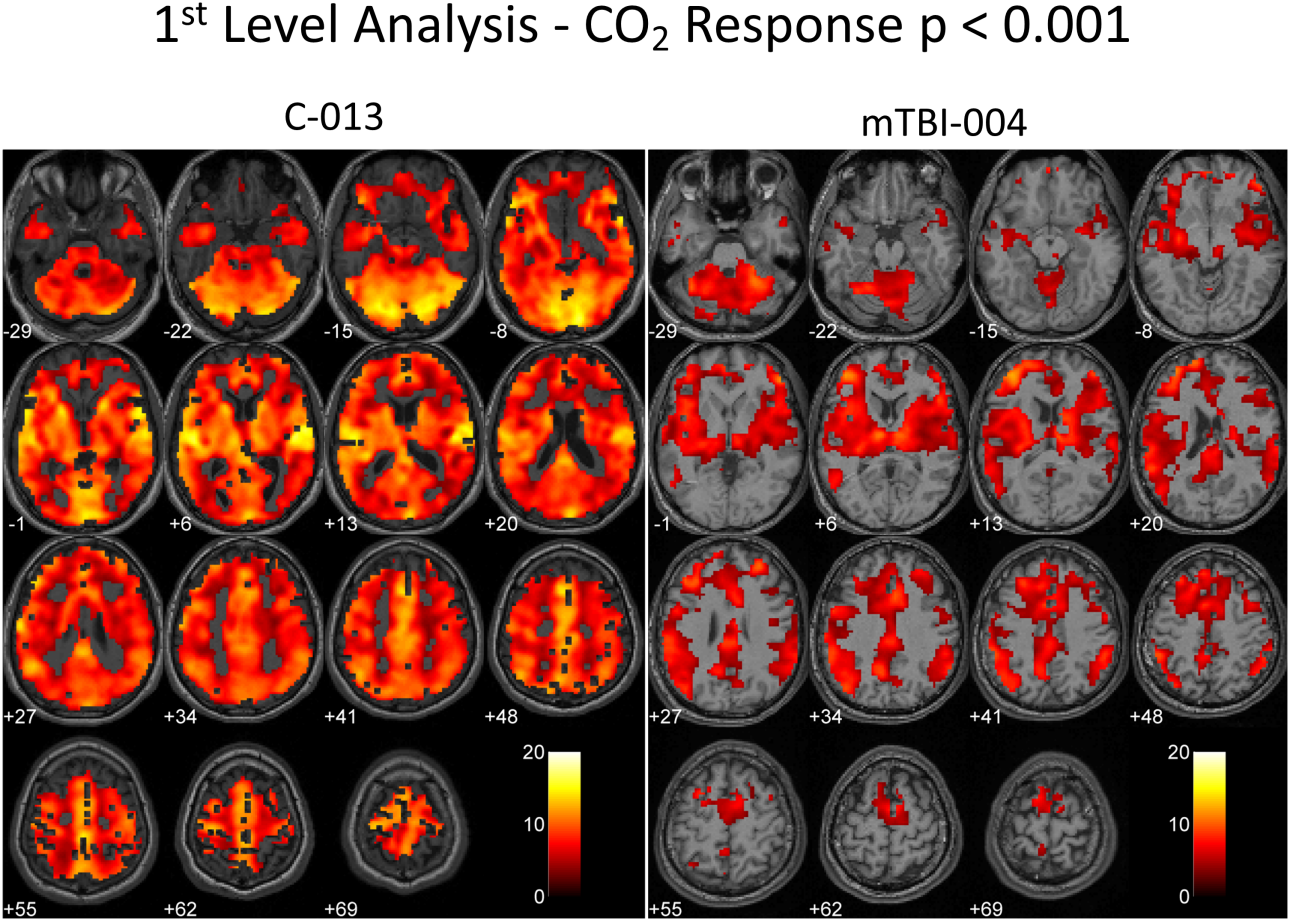
A and B: A 1^st^ level analysis of a control subject and a symptomatic PCS patient. The CO_2_ response was assessed at the p = 0.001 level in both. The number of voxels responding to CO_2_ in the control patient (C-013) was 94% of the total brain voxel count at this level of statistical significance. In the symptomatic PCS patient (mTBI-004) the voxel response ratio was 46%. The numbers at the left below each axial slice represent the level below or above the anterior commissure – posterior commissure (AC–PC) line. The numbers related to the colour bar are the t-values for the statistical parametric mapping (SPM) 1^st^ level analysis.

The 1^st^ level BOLD analysis is shown in Table 3. In the control subject group at the p<0.001 level there was an anticipated cerebrovascular response to CO_2_ in 72±21% of the voxels. The mean CO_2_ response in the symptomatic concussion patients was 63±15%; the mean CO_2_ response in the asymptomatic concussion patients was 48±37%; p = 0.307 between groups. Two representative examples of the response to CO_2_ are shown for a control subject (C-013) and a concussion patient (mTBI-004) in Figure 3A and B for the stress test.

**Table 3:** 1st Level Analysis.

	Controls	Sym PCS	Asym PCS	p-values
Voxel Count	56929±713	57318±1830	59059±928	0.092
Whole Brain CVR	0.36±0.03	0.34±0.06	0.38±0.05	0.447
R^2^-value	0.86±0.10	0.91±0.06	0.89±0.07	0.525
% Increased Response to CO_2_	72±21	63±15	47±38	0.307
% Decreased Response to CO_2_	0.02±0.03	1.3±1.5	0.07±0.08	0.084

PCS-post-concussion syndrome.

Sym-symptomatic.

Asym-asymptomatic.

Voxel Count in MNI space.

CVR - cerebrovascular reactivity in % arbitrary BOLD units/mmHg CO_2_.

% Increased - ratio responding:whole brain voxel count.

% Decreased - ratio responding:whole brain voxel count.

**Figure 4:**
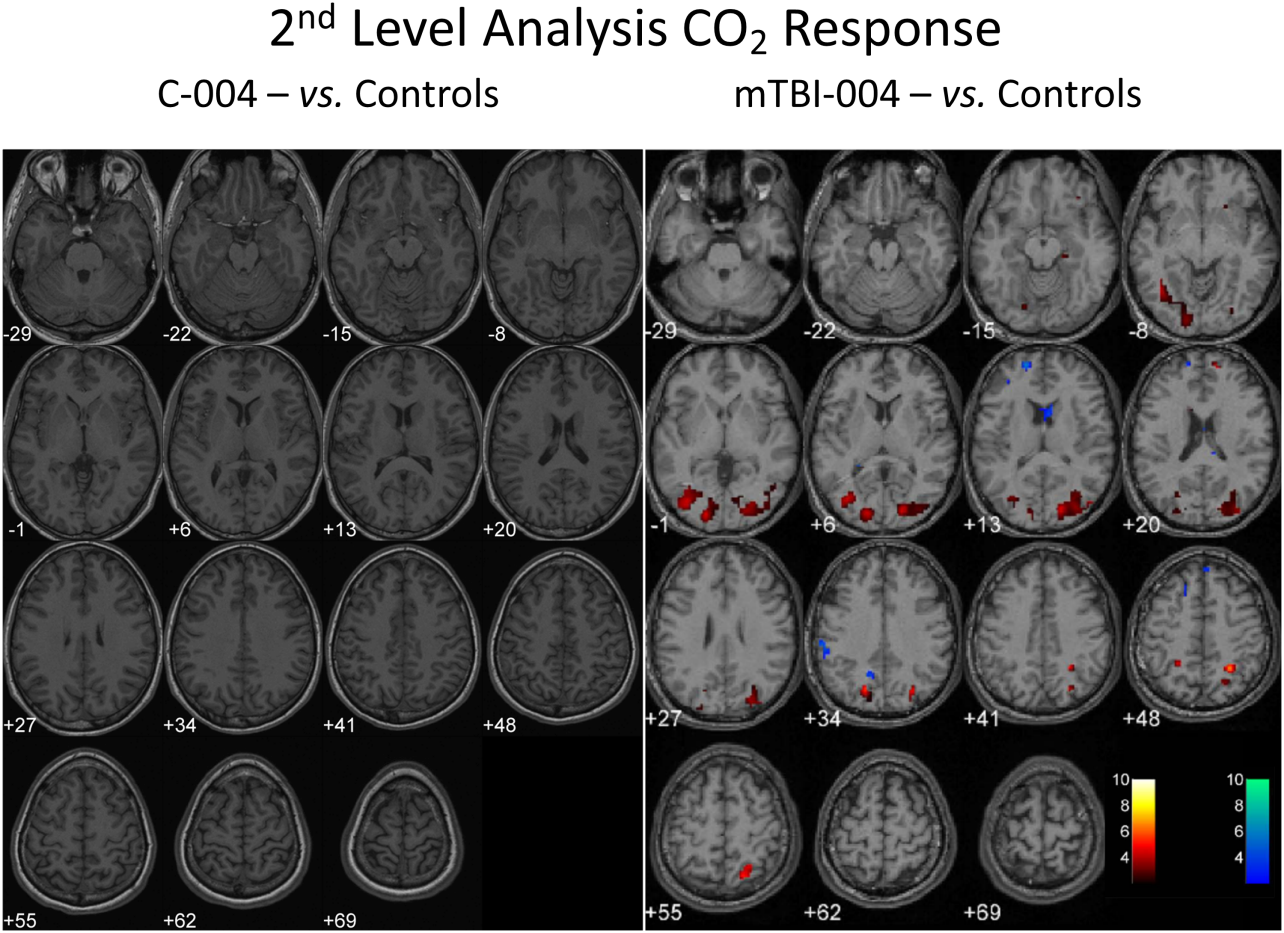
A and B: A 2^nd^ level analysis for a control subject (C-004) compared to the grouped control atlas and a symptomatic PCS patient (mTBI-004) compared to the same atlas of control subjects. The ‘hot’ color scale shows ‘blobs’ where the CO_2_ response was less than seen in the controls at the p = 0.05 level. The ‘cold’ colour scale shows where the CO_2_ response was greater than in the controls at the p = 0.05 level. The levels below and above the AC–PC line are the same as in Figure 3. The numbers related to the colour bar are the t-values for the SPM 2^nd^ level analysis.

The 2^nd^ level analysis is shown in Table 4. There were no voxels differing at the p = 0.05 level when any one control subject was compared to the group (see Figure 4A). In contrast a representative result from a symptomatic concussion patient is shown Figure 4B. The statistical analysis comparing any PCS patients to the control group collapses to a one-way t-test as there was a null response in the control group when comparing any control subject to the results in the group atlas. In the symptomatic PCS group the voxel ratio for decreased response to CO_2_ had p = 0.012 vs. zero mean and for the ratio of increased response to CO_2_ the p-value = 0.139. The respective results for asymptomatic concussion patients were p = 0.053 and 0.281.

**Table 4:** 2nd Level Analysis.

	Controls	Sym PCS	Asym PCS	p-values
Voxel Count	56929±713	57318±1830	59059±928	0.092
% Increased Response to CO_2_	0±0	3.01±5.10	4.44±6.78	0.378
% Decreased Response to CO_2_	0±0	1.97±1.64	0.49±0.31	0.024
% Combined	0±0	4.98±4.66	4.93±6.69	0.156

PCS-post-concussion syndrome.

Sym-symptomatic.

Asym-asymptomatic.

% Increased - ratio responding:whole brain voxel count.

% Decreased - ratio responding:whole brain voxel count.

% Combined - (% increased + % decreased) ratio responding:whole brain voxel count.

Regressing the CO_2_ voxel count ratios on the PCSS scores did not reveal statistical significance for the increased or decreased response to CO_2_ in either the symptomatic or asymptomatic patients.

## Discussion

There is a real need for novel neuro-imaging assessment tools that can be used to evaluate concussion in individual patients and generate useful quantitative biomarkers that can aid in the diagnosis, prognostication, and management of post-concussion syndrome (PCS). Recent studies have applied sophisticated neuro-imaging techniques including magnetoencephalography (MEG), diffusion tensor imaging (DTI) and tractography, and task-based fMRI [16] [17] [18] [19] [20] [21] [22] [23] [24] [25] [26] [27]. Despite detecting changes between groups of concussion patients and healthy controls, none of these techniques have emerged as clinical tools that can reliably confirm the diagnosis of concussion, quantify concussion severity, or assess clinical recovery in individual patients.

The brain is exquisitely sensitive to the arterial partial pressure of carbon dioxide (PaCO_2_), the most potent physiological stimulus of arterial dilatation, whereby each mmHg increase in PaCO_2_ is accompanied by a 2–15% increase in CBF. It is this increase in CBF with concomitant changes in cerebral oxygenation (measured indirectly by alterations in the regional BOLD signal) which occurs with controlled alterations in CO_2_ which is the foundation of the brain stress test described here. [28] [29] [30] [31] Given the acute changes in cerebral metabolism that occur during TBI and concussion [32], the inability of the brain to retain an appropriate CVR to CO_2_ may be an important mediator of persistent concussion symptoms and patient outcomes. Although the magnitude and temporal pattern of CBF and CO_2_ regulation impairments have an important predictive impact on patient outcomes in children with severe TBI [33] [34], the role of these cerebrovascular parameters in patients with concussion and post-concussion syndrome (PCS) has not been studied previously. Work in an animal model of concussion using two-photon laser scanning microscopy indicates that parenchymal damage occurs acutely with controlled compression of cerebral tissue. [35] Vascular disruption associated with astrocyte death and microglial invasion at the glial limitans is characteristic. These authors suggest that this signature, as seen in their murine model, is reflected clinically as gadolinium-enhanced meningeal thickening in up to 47% of concussed patients imaged with post-contrast fluid attenuated inversion recovery (FLAIR) MRI. Attwell and colleagues [36] have highlighted the relationship between the triad of neuron-astrocyte-endothelial cell to control regional cerebral blood flow. Astrocyte death and vascular disruption provide a means to explain altered CO_2_ responsiveness post-concussion. The mechanisms for abnormal or inverse responsiveness to a CO_2_ stimulus with a provocation test such as used in this study has also recently been documented [37].

In this pilot study, provocative CO_2_ challenge during BOLD MRI demonstrated abnormal cerebrovascular responsiveness to CO_2_ in concussion patients. Both a diminished and enhanced response to CO_2_ was observed when compared to the atlas of control subjects. The magnitude of hypo-responsiveness compared to control subject had a range from 0.1–4.7% of total voxels, while the magnitude of hyper-responsiveness ranged from 0.3–15.4% for the concussion patients. Despite these differences, whole brain BOLD CVR did not differ when concussion patients were compared to controls. This likely relates to the fact that the absolute magnitude of altered voxels was relatively low and in part masked by the fact that in each concussion patient there was a hypo and hyper response – negating potential differences from control subjects. The 2^nd^ level analysis revealed an abnormal CO_2_ response signature for each concussion patient when compared on an individual basis to the control atlas.

In addition to providing preliminary evidence of altered CVR to CO_2_, this report also introduces a novel neuro-imaging assessment tool that warrants further study in patients with acute concussion and PCS. Several features of this test may offer advantages to alternative neuro-imaging measures based on task-based fMRI paradigms utilized in concussion. In contrast to other imaging methods used to measure CO_2_ responsiveness including single photon emission tomography, xenon-enhanced computerized tomography, and positron emission tomography this technique does not require the use of radiation or intravenous radio-labeled tracers or contrast agents. As well, unlike concussion-specific symptom inventories, computerized neurocognitive instruments and task-based fMRI techniques that depend greatly on the motivated self-reporting or performance of the patient, the MPET technique generates a highly reproducible stimulus, requiring only a small increased work of breathing on the part of the patient. This technique does not allow patients, especially athletes, to “fake” or “sandbag” baseline studies nor mask the detection of clinically meaningful data through a lack of effort or self-reporting during post-injury studies. We also believe the activation by a CO_2_ stimulus as employed in this test results in hyperventilation, as occurs with exercise, and therefore is relevant to those athletes whom are symptomatic post-concussion with exertion, in comparison to the simple motor and memory tasks used in task-based fMRI studies that do not resemble any sport-specific activities. Finally, task-based fMRI studies that rely on group comparisons to generate results provide no quantifiable indices that can be followed throughout recovery; in contrast this assessment tool generates biomarkers that can be compared to results from age- and gender-matched controls, individual patient baseline studies, and against concussion symptom inventory and neuro-cognitive testing scores.

### Limitations of the current study

This study represents a pilot of the feasibility of the methodology presented. The ability of an MRI brain CO_2_ stress test to accurately gauge severity and prognosis and confirm recovery following concussion will only be fully determined with additional longitudinal studies. As described the test was sensitive – with a 100% incidence of at least some voxels present with both an enhanced and diminished CO_2_ responsiveness seen with both symptomatic and asymptomatic concussion patients when compared to healthy control subjects. What represents an appropriate ‘cut-point’ for the minimum ratio of voxels with diminished CO_2_ response: whole brain voxel count, by way of example, will require further study. In theory, the MPET device is well suited to permit such an approach as each patient’s physiological parameters are entered to permit control of end-tidal gases. These parameters are stored and can be accessed for use in follow-up studies. As a concussion is uniquely individualized, longitudinal study of each patient may be the most valuable approach for assessment. The MRI CO_2_ stress test is not inexpensive, as the assessment requires access to a 3.0 T magnet, with onsite availability of an individual trained to use the MPET device. Various other approaches to alter end-tidal CO_2_ can be utilized, but with limitations. [38] Other approaches to measuring the response to CO_2_ in the brain are available such as cerebral oximetry and transcranial Doppler. A marriage of these two techniques could potentially provide a surrogate test without the need for an MR scan. Other limitations related to MR scanning specific to the CO_2_ stress test include patient acceptance – we had 3 out of 30 subjects with a claustrophobic/hypercapnic response requiring cessation of the test. We are working to limit this dropout rate by adjusting the upper limit of the hypercapnic stimulus. Excessive movement during scanning can also be a problem – especially as increased CO_2_ enhances the drive to breathe with translation and pitch in the z-plane during hyperventilation. Optimizing patient positioning with padding around the shoulders and neck can help here as well as reducing the magnitude of the change in CO_2_ levels for the stress test.

In conclusion we report on an MRI CO_2_ brain stress test, which may aid in the diagnosis, prognosis and management of patients suffering from concussion. We believe the greatest advantage offered by our novel approach is that the CO_2_ stress test represents a repeatable, dynamic assessment of brain function that provides quantitative biomarkers that can be followed over time.
